# Differential expression of microRNAs in tomato leaves treated with different light qualities

**DOI:** 10.1186/s12864-019-6440-4

**Published:** 2020-01-13

**Authors:** Fei Dong, Chuanzeng Wang, Yuhui Dong, Shuqin Hao, Lixia Wang, Xiudong Sun, Shiqi Liu

**Affiliations:** 1 Vegetable and Flower Research Institute of Shandong Academy of Agricultural Sciences / Shandong Key Laboratory of Greenhouse Vegetable Biology / Shandong Branch of National Vegetable Improvement Center / Vegetable Science Observation and Experimental Station in Huang-Huai District of the Ministry of Agriculture, Jinan, 250100 China; 2 College of Horticulture Science and Engineering, Shandong Agricultural University, Tai An, 271018 China; 3 Shandong Academy of Agricultural Sciences, Jinan, 250100 China; 4 Shandong Agriculture and Engineering University, Jinan, 250100 China; 5 Shenyang Agriculture University, Shenyang, 110866 China; 6 State Key Laboratory of Crop Biology, Tai An, 271018 China; 7 Ministry of Agriculture Key Laboratory of Biology and Genetic Improvement of Horticultural Crops in Huanghuai Region, Tai An, 271018 China

**Keywords:** Tomato, Light quality, MicroRNA, Target gene, Plant hormone

## Abstract

**Background:**

Light is the main source of energy and, as such, is one of the most important environmental factors for plant growth, morphogenesis, and other physiological responses. MicroRNAs (miRNAs) are endogenous non-coding RNAs that contain 21–24 nucleotides (nt) and play important roles in plant growth and development as well as stress responses. However, the role of miRNAs in the light response is less studied. We used tomato seedlings that were cultured in red light then transferred to blue light for 2 min to identify miRNAs related to light response by high-throughput sequencing.

**Results:**

A total of 108 known miRNAs and 141 predicted novel miRNAs were identified in leaf samples from tomato leaves treated with the different light qualities. Among them, 15 known and 5 predicted novel miRNAs were differentially expressed after blue light treatment compared with the control (red light treatment). KEGG enrichment analysis showed that significantly enriched pathways included zeatin biosynthesis (ko00908), homologous recombination (ko03440), and plant hormone signal transduction (ko04075). Zeatin biosynthesis and plant hormone signal transduction are related to plant hormones, indicating that plant hormones play important roles in the light response.

**Conclusion:**

Our results provide a theoretical basis for further understanding the role of miRNAs in the light response of plants.

## Background

Tomato (*Solanum lycopersicum* L.) is one of the main vegetables under protected cultivation and is an important part of the human diet. Tomato is also a model plant that has long been used in studies of plant genetics, development, physiology, pathology, and fleshy fruit ripening. Therefore, a lot of biological information about this important economic crop has accumulated [1].

MicroRNAs (miRNAs) are a class of endogenous non-coding RNAs that regulate gene expression at the post-transcriptional level by binding to complementary regions of the mRNAs of their target genes to degrade or inhibit the mRNAs [2, 3]. Mature miRNAs are 21–24 nt long and are derived from pre-miRNAs that undergo two cleavage steps catalyzed by Dicer-like 1 (DCL1) to produce miRNA:miRNA* double strands, which then form mature miRNAs after uncoiling [4]. MiRNAs not only regulate the development of leaf, flower, and fruit in tomato [5–9], but also are involved in the biotic and abiotic stress responses of tomato plants [2, 4, 10–13]. However, there are few reports on the roles of miRNAs in the light response.

Light is the main source of energy and is one of the most important environmental factors for plant growth, morphogenesis, and other physiological responses [14–16]. Light regulates germination, de-etiolation, phototropism, flowering, leaf and stem growth, biological clock, stomatal opening, chloroplast relocation, and anthocyanin synthesis in plants [17, 18]. Light quality is an important aspect of light research because it affects plant morphology and yield [16, 19], as well as plant quality [19, 20]. Light quality regulates plant growth and development through various photoreceptors which stimulate signal transduction systems to change plant morphology [19, 21]. Among them, red light can promote the growth of plants by promoting the elongation of hypocotyls, while blue light mainly inhibits the elongation and induction of hypocotyls [22, 23]. Blue light increases the net photosynthetic rate of tomato seedling leaves by inducing stomatal opening [24]; while red light reduces stomatal conductance and increases intercellular CO_2_ concentration, resulting in a decrease in photosynthetic rate [25]. In addition, red light inhibits the photosynthetic product from the leaves, which increases the starch accumulation of the leaves. The excessive accumulation of starch is not conducive to the photosynthesis of plant leaves [26].

The effects of light quality on plant miRNAs have been reported for soybean seedlings [27], callus of longan [28], *Brassica rapa L. subsp*. rapa cv. Tsuda [29], *Arabidopsis thaliana* [30, 31], and wheat [32]. However, the effect of different light qualities on the miRNAs of tomato seedlings has not been reported so far. In this study, we cultured tomato seedlings in red light then transferred some of them to blue light for 2 min, and identified miRNAs related to the light signal response by high-throughput sequencing. The results will provide a theoretical foundation for further understanding the role of miRNAs in the light response of plants.

## Results

### Sequence analysis, and classification and annotation of the small RNAs (sRNAs)

A total of 67,554,747 and 69,317,206 raw reads were obtained from the leaves of tomato plants treated with red light (control) and blue light (treated), respectively. After removing the low-quality reads, 58,657,965 (87.1% of the total) and 59,204,048 (85.4% of the total) clean reads were obtained in the red light and blue light libraries, respectively (Table 1).

**Table 1:** Quality control of the clean reads data

Sample	Raw reads	Clean reads	Raw clean reads %	Low quality %	Containing ‘N’ reads	Length < 18	Lenghth> 30	Q30%
S01	27,670,941	23,487,978	84.88	0	0	2,173,796	2,009,167	98.46
S02	19,651,152	17,574,787	89.43	0	0	837,356	1,239,009	98.70
S03	20,232,654	17,595,200	86.96	0	0	1,104,045	1,533,409	98.67
S04	22,329,405	18,822,750	84.30	0	0	1,633,140	1,873,515	98.69
S05	25,943,329	22,307,050	85.98	0	0	1,236,511	2,399,768	98.51
S06	21,044,472	18,074,248	85.89	0	0	650,390	2,319,834	98.49

Note: S01, S02, S03 are red-treated tomato leaves, S04, S05, and S06 are blue-treated tomato leaves

Note: S01, S02, S03 are red-treated tomato leaves, S04, S05, and S06 are blue-treated tomato leaves.

**Fig. 1:**
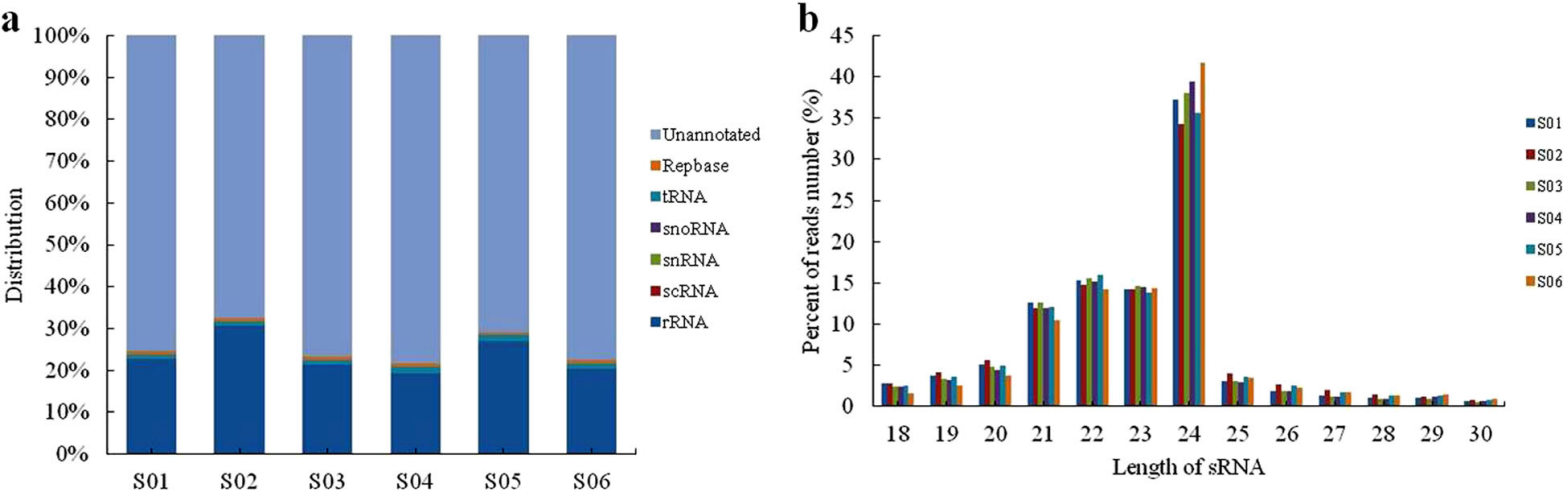
Analysis of the small RNAs (sRNAs) in the blue light and red light treated libraries. **a** Classification of the sRNA sequences in the two libraries. **b** Length distribution of sRNA in the two libraries. S01, S02, S03 are red light treated tomato leaves; S04, S05, and S06 are blue light treated tomato leaves

Most of the sRNAs in the red light and blue light libraries were unannotated, followed by those identified as rRNAs. Only a small proportion of the sRNAs were identified as scRNAs, snRNAs, snoRNAs, or tRNAs (Fig. 1a). Generally, sRNAs are 18–30 nt long, and in the control and treated libraries, most of them were 24 nt long, accounting for 36.57 and 38.90% of the total reads, respectively. Furthermore, most of the reads (90.16 and 89.52%) in the control and treated libraries were ≤ 24 nt (Fig. 1b).

### Identification of known miRNA

We identified 108 known miRNAs in the two libraries, and 88 of them were from 39 miRNA families. The numbers of miRNAs in each family varied significantly; the most abundant family was MIR482 with 7 members, followed by the MIR171_1 and MIR156 families with 5 members each. Twenty families (MIR477, MIR833, MIR6023, MIR1120, MIR397, MIR399, MIR1516, MIR319, MIR4376, MiR6024, MIR5303, MIR160, MIR5304, MIR6022, MIR6026, MIR162_1, MIR8167, MIR169_2, MIR5208, and MIR5160) had only one member (Additional file 1: Table S1). The expression levels of different miRNAs varied significantly with counts ranging from 1 to 181,400. Sly-miR159 was the most abundant with 137,039 and 129,853 counts in the control and treated libraries, respectively (Additional file 2: Table S2).

### Prediction of novel miRNAs

We identified 141 candidate novel miRNAs in the two libraries, and 79 of them were from 56 miRNA families. The most abundant family was MIR398 with 9 members, followed by the MIR5303 and MIR400 families with 4 members each (Additional file 3: Table S3). Among the novel miRNAs, unconservative_1_3796 was the most abundant with 101,523 and 100,601 counts in the control and treated libraries, respectively (Additional file 2: Table S2).

### Differential expression of miRNAs

**Fig. 2:**
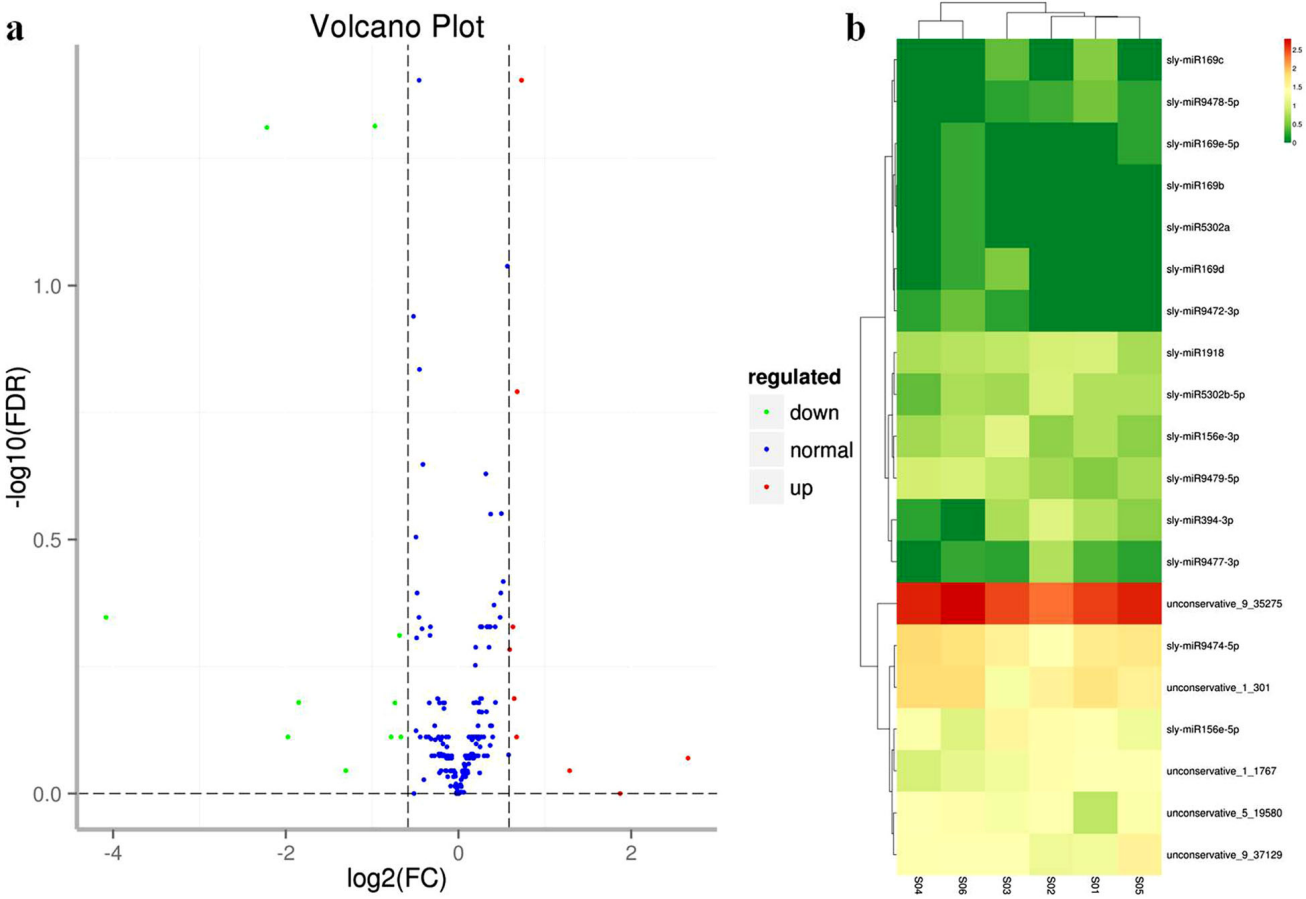
Differentially expressed miRNA between the red light and bule light treated libraries. **a** Scatter diagram of the differential read counts of miRNA. Each point in the figure represents a miRNA. Blue points indicate miRNAs that were not differentially expressed; red points indicate up-regulated miRNAs; green points indicate down-regulated miRNAs. **b** Heat map of the differentially expressed miRNAs. Columns represent different leaf samples; rows represent different miRNAs. The clustering was performed using the log10 (TPM + 1) values. Red indicates highly expressed miRNAs; green indicates lowly expressed miRNAs

Overall, we identified 249 miRNAs (108 known and 141 novel) in the two libraries. By comparing the expression levels of the miRNAs between the red light treated (control) and blue light treated libraries we identified 15 known and 5 novel miRNAs that were differentially expressed. Among them, 10 miRNAs (sly-miR169b, sly-miR169e-5p, sly-miR5302a, sly-miR9472-3p, sly-miR9474-5p, sly-miR9479-5p, unconservative_1_301, unconservative_5_19580, unconservative_9_35275, and unconservative_9_37129) were up-regulated, and 10 miRNAs (sly-miR156e-3p, sly-miR156e-5p, sly-miR169c, sly-miR169d, sly-miR1918, sly-miR394-3p, sly-miR5302b-5p, sly-miR9477-3p, sly-miR9478-5p, and sly-miR9478-5p) were down-regulated (Fig. 2; Additional file 4: Table S4).

### Prediction and functional analysis of the target genes of the differentially expressed miRNAs

**Fig. 3:**
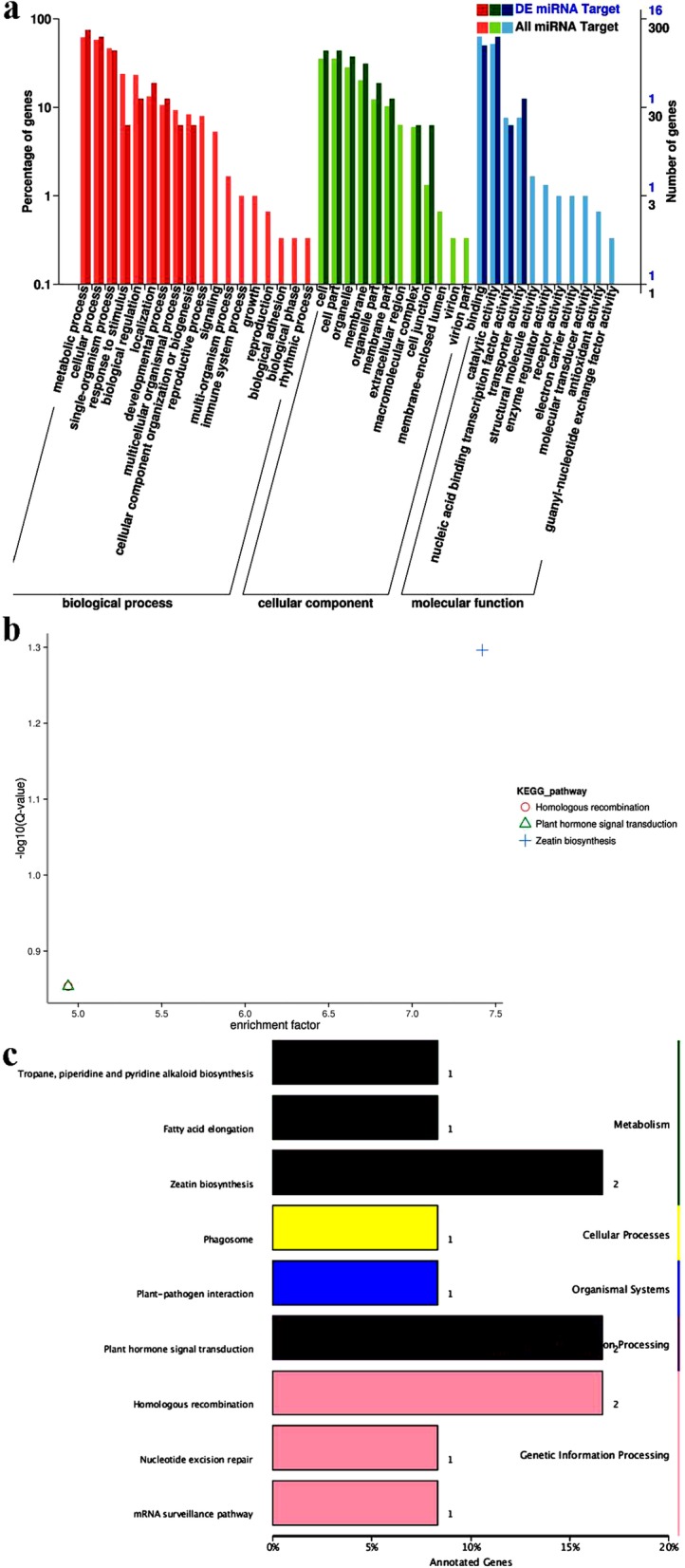
Gene ontology (GO) classification and kyoto encyclopedia of genes and genomes (KEGG) analysis of target genes of the differentialy expressed miRNAs. **a** GO annotation of 16 candidate target genes. The x-axis indicates the GO category; the right y-axis indicates the number of target genes in a category; the left y-axis indicates the percentage of target genes annotated with a specific term under the main category. **b** KEGG pathway enrichment scatter plot of 17 candidate target genes. Each graph represents a KEGG pathway and the pathway name is shown in the right graph. The abscissa is the enrichment factor, indicating the proportion of the number of differentially expressed miRNA target genes annotated to a certain pathway in the total number of genes annotated to this pathway. The larger the enrichment factor, the more significant the level of enrichment of differentially expressed miRNA target genes in this pathway. The ordinate is -log10 (Q value), where Q is the *P* value after correction by the multiple hypothesis test. Thus, the larger the ordinate, the more reliable the significance of the enrichment of the differentially expressed miRNA target gene in this pathway. **c** KEGG classification map of the target genes of the differentialy expressed miRNAs. The ordinate is the KEGG metabolic pathway; the abscissa is the number of genes annotated to the pathway and their proportion to the total number of genes annotated

A total of 40 target genes of the 20 differentially expressed miRNAs were identified and functionally annotated with gene ontilogy (GO) terms and kyoto encyclopedia of genes and genomes (KEGG) pathways. Among them, 16 and 17 target genes were assigned GO terms and KEGG pathways, respectively. The GO analysis showed that under the biological process, cell component, and molecular function categories, metabolic process (GO: 0008152), membrane (GO:0016020), and catalytic activity (GO: 0003824) was the most enriched terms, respectively (Fig. 3). In the blue light treated library, DNA metabolic process (GO:0006259), bounding membrane of organelle (GO:0098588), and nucleic acid binding (GO:0003676) were significantly enriched (Additional file 5: Table S5).

The KEGG analysis showed that the 17 annotated target genes were associated with 9 metabolic pathway, and pathways that contained several target genes were obviously enriched, namely zeatin biosynthesis (ko00908), homologous recombination (ko03440), and plant hormone signal transduction (ko04075) (Fig. 3, Additional file 6: Table S6).

### Verification of differentially expressed miRNAs and target genes

**Fig. 4:**
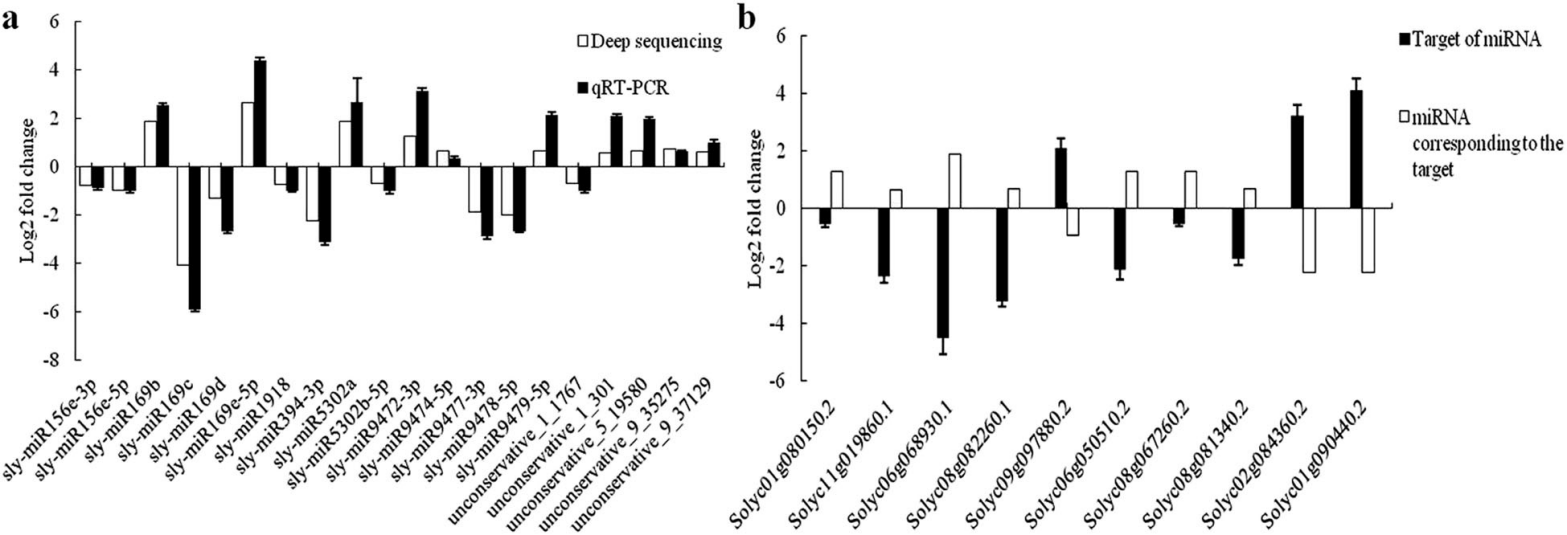
Verification of the differentially expressed miRNAs and their target genes by qRT-PCR. **a** Verification of differentially expressed miRNAs. **b** Verification of the target genes target genes in tomato. Each bar represents the mean ± standard errors of triplicated assays

The expression levels of the 20 differentially expressed miRNAs and 10 randomly selected target genes were verified by quantitative reverse transcription PCR (qRT-PCR). The expression patterns of the miRNAs determined by qRT-PCR were consistent with those from the Illumina RNA sequencing data, and the expression patterns of the 10 target genes were opposite to those of the corresponding miRNAs (Fig. 4). For example, target genes Solyc06g068930.1 and Solyc08g082260.1 were down-regulated under blue light, whereas their corresponding miRNAs were up-regulated (Fig. 4b).

## Discussion

As non-coding small RNAs, miRNAs play important roles in plant growth and development, and stress response [2, 4, 8, 11, 13]. Previous studies have shown that miRNA regulate many stress-related genes, including those encode dehydrins, late embryo abundant proteins (LEA), glutathione S-transferase (GST), transcription factors (GRAS, ARF and MYB), and hormonal pathway components [4]. With the development of next-generation sequencing technology, high-throughput sequencing has been widely used to identify conserved and novel miRNAs in plants [4, 33, 34]. However, there are few reports about the effect of light quality on tomato miRNAs. In addition, studies have shown that the earliest significant changes we detected in almost all tissue types sampled initiated at 1 or 2 min post high light stress application to the local leaf [35]. Therefore, in this study, tomato seedlings were cultured in red light then some were transferred to blue light for 2 min. Then the red light treated (control) and blue light treated libraries were compared to detect miRNAs related to light signals.

The sequencing results showed that 24 nt long sRNAs were the most abundant in the control and blue light treated libraries, accounting for 36.57 and 38.90% of the total reads respectively. This finding is consistent with previous studies in tomato [2, 4, 8, 11]. It has been reported that 24 nt sRNAs play important roles in transcriptional silencing of transposon and pericentromeric regions through RNA-directed DNA methylation [11].

We detected 249 miRNAs in the two libraries. Of the 108 known miRNAs, 88 belonged to 39 families. The number of miRNAs in each family varied greatly, with the MIR482 family having the most members. The expression levels of different miRNAs also varied greatly, with counts ranging from 1 to 181,400. Sly-miR159 was the most highly expressed, which is consistent with previous results [4, 36]. Of the 141 novel miRNAs, 79 belonged to 56 families, and the MIR398 family had the most members. Unconservative_1_3796 was the most highly expressed of the novel miRNAs, having 101,523 and 100,601 counts in the red light control and blue light treated libraries, respectively (Additional file 2: Table S2).

The important regulatory function of miRNAs in controlling the expression of photoresponse genes has been reported previously. For example, Pashkovskiy et al. [37] reported that blue light significantly increased the expression of miR167, which reduced the expression of its target genes *ARF6* and *ARF8*, thereby reducing the activation of auxin-dependent genes. Zhou et al. [29] showed that during the development of *Brassica rapa subsp. Rapa* cv. Tsuda seedlings, blue light specifically down-regulated miR156 and miR157, and up-regulated the target genes *SPL9* and *SPL15*. Li et al. [38] reported that blue light inhibited the expression of miR394, which promoted the expression of the target genes and the accumulation of flavonoids and epicatechins, but inhibited the synthesis of rutin. We found that sly-miR156e-3p, sly-miR156e-5p, and sly-miR394-3p were significantly down-regulated in the blue light treated leaves, which is consistent with the above research results. In addition, 20 miRNAs (15 known and 5 novel) belonging to 8 families (MIR169_2, MIR169_1, MIR5302, MIR1516, MIR156, MIR394, MIR837, and MIR8005) were differentially expressed in the blue light treated leaves; 10 were up-regulated (sly-miR169b, sly-miR169e-5p, sly-miR5302a, sly-miR9472-3p, sly-miR9474-5p, sly-miR9479-5p, unconservative_1_301, unconservative_5_19580, unconservative_9_35275, and unconservative_9_37129) and 10 were down-regulated (sly-miR156e-3p, sly-miR156e-5p, sly-miR169c, sly-miR169d, sly-miR1918, sly-miR394-3p, sly-miR5302b-5p, sly-miR9477-3p, sly-miR9478-5p and unconservative_1_1767).

Plant hormones are important regulators of plant growth, development, and stress responses [4, 39]. Previous studies have shown that plant hormone signal transduction pathways were involved in the growth and development of sugarcane [36], maize [40], and radish [41], as well as in the responses to pathogenic microorganism infection [42–44], salt stress [45], and drought [46, 47]. In this study, the KEGG enrichment analysis showed that plant hormone signal transduction pathways not only contained more target genes, but also were obviously enriched. Our results showed that the differentially expressed miRNAs sly-miR169b and sly-miR9474-5p targeted genes that encode TIFY protein and protein phosphatase 2C (PP2C), respectively, which are involved in plant hormone signal transduction pathways (Additional file 6: Table S6). TIFYs are plant-specific transcription factors that are encoded by multiple genes and are highly conserved, especially the core motif TIF [F/Y] XG [48, 49]. Members of the TIFY protein family are involved in many biological processes. For example, overexpression of *AtTIFY1* (also known as *ZIM*) in *Arabidopsis thaliana* led to the extension of the petiole and hypocotyls [50], overexpression of *AtTIFY4a* (also known as *PPD1*) and *AtTIFY4b* (also known as *PPD2*) contributed to the synchronous growth of *Arabidopsis* leaves [51], and overexpression of *JAZ* affected the response to biotic and abiotic stresses through mediating jasmonic acid signal transduction [52–55]. PP2C is a protein serine/threonine phosphatase and a key enzyme in the regulation of reversible protein phosphorylation. PP2C is present as a monomer in cells and its catalytic activity is Mg^2+^ or Mn^2+^ dependent. Reversible phosphorylation catalyzed by protein kinases and protein phosphatases is an important component of signal transduction, which is required for almost all physiological and pathological processes. In plants, PP2C is closely related to signal transduction [56], growth and development [57]; response to abiotic stresses such as drought [58], low temperature [59], high salt [60], and mechanical damage [61]; biotic stresses such as bacterial infection [62].

Zeatin is a cytokinin that plays an important role in cell division. The blue light responding sly-miR9472-3p foung in this study targeted the gene encoding adenylate isopentenyltransferase 5, which is associated with the zeatin biosynthesis signaling pathway (Additional file 6: Table S6). Adenylate isoamyltransferase, a rate-limiting enzyme in cytokinin synthesis, catalyzes the transfer of the isopentenyl group of dimethylallyl pyrophosphate to the amino terminal N^6^ of ATP, ADP, and AMP. In addition to their catalytic function, some adenylate isoamyltransferases have stress resistance functions. In tomato plants, two isopentenyltransferases (SlIPT3 and SlIPT4) were found to be involved in the response to salt stress [63]. In *Clematis paniculata*, adenylate isopentenyltransferase was associated with responses to external stresses (UV-B, salt stress, and drought) [64]. The reduction of endogenous cytokinins in *Arabidopsis* was shown to enhance plant salt and cold tolerance [65]. We suggest the up-regulation of sly-miR9472-3p may inhibit the activity of adenylate isoamyltransferase, thereby decreasing cytokinin levels and enhancing the plant tolerance. Zeatin biosynthesis and plant hormone signal transduction are both signaling pathways related to plant hormones, which indicates that plant hormones play very important roles in the light response of plants.

## Conclusions

We identified 108 known miRNAs and 141 candidate novel miRNAs. Among them, 15 known and 5 novel miRNAs were differentially expressed in tomato leaves after blue light treatment. KEGG enrichment analysis of the target genes revealed that the zeatin biosynthesis (ko00908), homologous recombination (ko03440), and plant hormone signal transduction (ko04075) pathways were obviously enriched. Zeatin biosynthesis and plant hormone signal transduction both are related to plant hormones, which indicates plant hormones play a very important role in the light response. Our results provide a theoretical basis for further understanding the role of miRNAs in light response.1.

## Methods

### Test materials and treatments

We used tomato *Solanum lycopersicum* L. cv Micro-Tom (Pan American Seed, USA) as the test material. Germinated seeds were sown in a substrate containing vermiculite and peat (1:2, V:V), then grown in a greenhouse. When the second true leaf had fully expanded, the same growth conditions were selected and transferred to the light quality laboratory of Scientific and Technological Innovation Park in Shandong Agriculture University and grown in the same substrate under red light (657 nm) until the five-leaf stage. Then, half of the seedlings were transferred to blue light (457 nm) for 2 min. The seedlings treated with red light were used as the control. The second leaf from the bottom of the tomato seedlings treated with red and blue light was removed and frozen in liquid nitrogen, then stored at − 80 °C for further experiments.

The LED light source was provided by Guangdong Chunying Photoelectric Technology Co., LTD. The light treatment scaffold was made of steel, and the light source was placed on the top. The scaffold was covered with silver shading cloth to ensure the LED light was the only light source for plant growth. Each treatment was completed with 50 seedlings, and all experiments were conducted in triplicate; the plants were arranged randomly. Photosynthetic photon flux density (PPFD) at 50 cm from the light source was 300 μmol∙m^− 2^∙s^− 1^, tomato plants were incubated under a 12-h light (28 °C):12-h dark (18 °C) photoperiod, and the relative humidity was 70% ± 10%.

### MiRNA isolation, library construction, and Illumina sequencing

Total RNA was extracted from the tomato leaves using a Trizol kit (Invitrogen, CA, USA) and purified using a miRNeasy Mini kit (Qiagen, Germany). The concentration and quality of the RNA samples were detected using a Nanodrop 2000 spectrophotometer (Thermo Fisher scientific, Wilmington, USA). The RNA libraries were constructed using a NEB Next Ultra small RNA Sample Library Prep Kit for Illumina strictly according to the manufacturer’s instructions. For each qualified RNA sample, 1.5 μg of RNA was supplemented to 6 μL by water, then the library was constructed using a small RNA Sample Preparation Kit. Adapters were added to the 5′ (phosphate group) and 3′ (hydroxyl group) ends of the sRNAs by T4 RNA ligase 1 and T4 RNA ligase 2 (truncated), respectively. The sequences with adaptors were used for cDNA synthesis and PCR amplification. The target fragments were screened by gel purification, and the fragments recovered from the gel were used to establish the sRNA libraries. The concentration of the sRNAs in each library was detected using Qubit 2.0, then they were diluted to 1 ng. μL^− 1^. The insert size was detected using an Agilent 2100 Bioanalyzer and the effective concentration of each library was measured by qRT-PCR to ensure the quality of the library. The qRT-PCR program was as follows: 95 °C for 5 min; 35 cycles of 95 °C for 30 s, 60 °C for 45 s, and 95 °C for 5 s. High-throughput sequencing of the libraries was carried out using an Illumina’s HiSeq X-Ten system, with single-end read length of 50 nt.

### Sequencing data analysis

The raw sequencing data were filtered to obtain clean reads for bioinformatics analysis. First, reads with 10% or higher unknown N bases and reads without the 3′ adaptor or insert sequences were removed. Then, the 3′ adaptor sequences and reads < 18 nt or > 30 nt were removed.

Bowtie [66] was used to BLAST the clean reads against four databases: SILVA, GtRNAdb, Rfam, and Repbase. Candidate miRNAs were obtained by filtering out reads that were identified as ribosomal RNA (rRNA), translocation RNA (tRNA), small nuclear RNA (snRNA), small nucleolar RNA (snoRNA), or repetitive sequences.

### Identification of known and novel miRNAs

We used the miRDeep2 (v2.0.5) package [67] to compare reads that were aligned to the tomato reference genome with known miRNA precursor sequences in the miRBase database. Reads that were identical to sequences in miRBase were considered to be known miRNAs. Potential miRNA precursor sequences were obtained by aligning the reads to the tomato genome sequence. Reads that did not find matches in miRBase were identified as novel miRNAs by Bayesian model grading based on the location of the reads in the precursor sequence (including mature, star, and loop) and the energy of the precursor structure determined by RNAfold randfold. The re-parameterized miRDeep2 is invoked. The length of sequences for predicting RNA secondary structure is set to 250, and a plant-specific scoring system is added to miRDeep2 [67]. Although miRDeep2 has been used mainly to identify animal miRNAs [67], it has been used to identify plant miRNAs after adjusting the parameters and grading system [68].

### Screening of differentially expressed miRNAs

MiRNA expression in each library was normalized using the two-phase multi-cast (TPM) algorithm [69] as follows: TPM = number of reads that mapped to one miRNA × 1,000,000 / number of reads that mapped to all miRNAs. The differentially expressed miRNAs between the two libraries were obtained using DESeq [70] with the screening criteria set as |log2(FC)| ≥ 0.584962500721156 and False discovery rate (FDR) ≤1. The fold change (FC) indicates the ratio of the expression levels in the two libraries.

### Prediction of miRNA target genes, and GO and KEGG pathway functional analysis

TargetFinder was used to predict the miRNA target genes [71]. The functions of the target genes were predicted by BLAST searches against the GO [72] and KEGG [73] databases.

### Verification of miRNAs and target genes by qRT-PCR

The expressions of 20 of the differentially expressed miRNAs and the corresponding 10 major target genes were detected by qRT-PCR. A SYBR® PrimeScript™ miRNA RT-PCR kit (TaKaRa) was used to produce the cDNA. Specific primers for the target genes were designed using Beacon Designer 7.9 (Table 2). The qRT-PCR cycle was as follows: preheat at 95 °C for 30 s; 40 cycles of denaturation at 95 °Cfor 20 s, then annealing at 60 °C for 20 s. Amplification curve and melting curve analyses (95 °C for 60 s, 55 °C for 30 s, and 95 °C for 30 s) were carried out to ensure the specificity of the products. All reactions were repeated three times. U6 and Actin4 (*Solyc04g 011500*) were used as the internal reference genes, and all data were analyzed using the 2^−△△Ct^ method.

**Table 2:** Primers used for qRT-PCR verification of the miRNAs and target genes

Primer name	Sequence (5′-3′)	Primer name	Sequence (5′-3′)
sly-miR156e-3p	GCTTACTCTCTATCTGTCACC	Solyc01g080150.2-F	TCCTCCTTCATCGTCAAC
sly-miR156e-5p	TAGCCAAGGATGACTTGCCTG	Solyc01g080150.2-R	AGAGTAGTAGTTCCATTCTTCC
sly-miR169b	CAGCCAAGGATGACTTGCCGA	Solyc11g019860.1-F	CAAGCGTAATACTCGTATGT
sly-miR169c	TAGCCAAGGATGACTTGCCTA	Solyc11g019860.1-R	GCTTCGTCAGTATCTTCTTC
sly-miR169d	TAGCCAAGGATGACTTGCCTTT	Solyc06g068930.1-F	TCAAGAATCTCGTCAACCT
sly-miR169e-5p	TGTTGGTGAGAGTTCGATTCTC	Solyc06g068930.1-R	GCAGCAGGAAGTGAAGTA
sly-miR1918	TGTTGGTGAGAGTTCGATTCTC	Solyc08g082260.1-F	GTAGTCGTGGTGTTGCTA
sly-miR394-3p	AGGTGGGCATACTGTCAACA	Solyc08g082260.1-R	CTGGCTCTGGTATCACAA
sly-miR5302a	AAACGAGGTTTGTTACTTTGG	Solyc09g097880.2-F	CTGGCTATTAGTGAGAAGAATC
sly-miR5302b-5p	TGAAATGCTATAGTTGGAAAGT	Solyc09g097880.2-R	GACGCATCTGTTCCAATAC
sly-miR9472-3p	TTCACAATCTCTGCTGAAAAA	Solyc06g050510.2-F	GAGAGGTGGCTGTGTTAT
sly-miR9474-5p	TGTAGAAGTCATGAATAAAATG	Solyc06g050510.2-R	CCAGGTGTAGAGTGTTGT
sly-miR9477-3p	TTGGGAAAGGGAACAACTGATAGT	Solyc08g067260.2-F	AGTAGAAGTTGGAGGAGATG
sly-miR9478-5p	GCTTAAATATGTAGATCGAACT	Solyc08g067260.2-R	AGACACAGAAGTGCTCAA
sly-miR9479-5p	TCCAGTCCTCTACCCTTCTCCA	Solyc08g081340.2-F	ATAGGATGGCAGGAGGTA
unconservative_1_1767	AAGAAAAACGACUGAAUAUAAAUC	Solyc08g081340.2-R	CAAGACGATGGTAAGACAAC
unconservative_1_301	AUUGCGAUGAUUAUGUUCAACCCU	Solyc02g084360.2-F	GCTTGATAACCGATGCTAG
unconservative_5_19580	AUGGUGUCUGGCUGUUAUUUCAGC	Solyc02g084360.2-R	GATGCGACTCATTATTGGAA
unconservative_9_35275	AUAUACAAAUUUCUGCUCAUUUCG	Solyc01g090440.2-F	GAGATGACTATGAAGAGGATGA
unconservative_9_37129	AUCUGUAUCUAGAAUUCAAAGUCU	Solyc01g090440.2-R	GAAGAGGATATTGGTGAGATTG
U6	AACAGTCTGACTTGTCCCTTC	Actin4-F	GAGGATATTCAGCCCCTTGTTTG
Universal reverse primer	GCATAGACCTGAATGGCGGTAAGG GTGTGGTAGGCGAGACATTTTTTTTTTTTTTTTTTTT	Actin4-R	CATCTTTCTGACCCATTCCAACC

## Supplementary information

**Additional file 1: Table S1.** Member numbers of known miRNAs in each miRNA family.

**Additional file 2: Table S2.** Count of miRNA in each sample.

**Additional file 3: Table S3.** Member number of Novel-miRNAs in each miRNA family.

**Additional file 4: Table S4.** The expression level of miRNA.

**Additional file 5: Table S5.** GO enrichment analysis.

**Additional file 6: Table S6.** KEGG analysis of target genes.

## Data Availability

The dataset supporting the conclusions of this article is available in the NCBI’s BioProject database [PRJNA523681].

## Abbreviations

DCL1: Dicer-like 1
FC: Fold change
FDR: False discovery rate
GO: Gene ontilogy
GST: Glutathione S-transferase
KEGG: Kyoto encyclopedia of genes and genomes
LEA: Late embryo abundant
miRNAs: MicroRNAs
nt: Nucleotide
PP2C: Protein phosphatase 2C
qRT-PCR: Quantitative reverse transcription PCR
rRNA: Ribosomal RNA
snoRNA: Small nucleolar RNA
snRNA: Small nuclear RNA
sRNAs: small RNAs
tRNA: Translocation RNA
